# Zika Virus Outbreak on Curaçao and Bonaire, a Report Based on Laboratory Diagnostics Data

**DOI:** 10.3389/fpubh.2019.00333

**Published:** 2019-11-12

**Authors:** Stephanie M. Lim, Robert Wever, Suzan D. Pas, Gygliola Bonofacio, Marion P. G. Koopmans, Byron E. E. Martina

**Affiliations:** ^1^Artemis One Health Research Foundation, Delft, Netherlands; ^2^Medical Laboratory Services, Willemstad, Curaçao; ^3^Department of Viroscience, WHO Collaborating Centre for Arboviruses and Hemorrhagic Fevers, Erasmus Medical Center, Rotterdam, Netherlands

**Keywords:** Zika virus, outbreak, laboratory, qRT-PCR, epidemiology, Curaçao, Bonaire

## Abstract

**Background:** Zika virus (ZIKV) emerged in May 2015 in Brazil, from which it spread to many other countries in Latin America. Cases of ZIKV infection were eventually also reported in Curaçao (January 2016) and Bonaire (February 2016).

**Methods:** In the period of 16 December 2015 until 26 April 2017, serum, EDTA-plasma or urine samples were taken at Medical Laboratory Services (MLS) from patients on Curaçao and tested in qRT-PCR at the Erasmus Medical Centre (EMC) in the Netherlands. Between 17 October 2016 until 26 April 2017 all samples of suspected ZIKV-patients collected on Curaçao, as well as on Bonaire, were tested at MLS. Paired urine and/or serum samples from patients were analyzed for ZIKV shedding kinetics, and compared in terms of sensitivity for ZIKV RNA detection. Furthermore, the age and gender of patients were used to determine ZIKV incidence rates, and their geozone location to determine the spatial distribution of ZIKV cases.

**Results:** In total, 781 patients of 2820 tested individuals were found qRT-PCR-positive for ZIKV on Curaçao. The first two ZIKV cases were diagnosed in December 2015. A total of 112 patients of 382 individuals tested qRT-PCR-positive for ZIKV on Bonaire. For both islands, the peak number of absolute cases occurred in November 2016, with 247 qRT-PCR confirmed cases on Curaçao and 66 qRT-PCR-positive cases on Bonaire. Overall, a higher proportion of women than men was diagnosed with ZIKV on both islands, as well as mostly individuals in the age category of 25–54 years old. Furthermore, ZIKV cases were mostly clustered in the east of the island, in Willemstad.

**Conclusions:** ZIKV cases confirmed by qRT-PCR indicate that the virus was circulating on Curaçao between at least December 2015 and March 2017, and on Bonaire between at least October 2016 and February 2017, with peak cases occurring in November 2016. The lack of preparedness of Curaçao for the ZIKV outbreak was compensated by shipping all samples to the EMC for diagnostic testing; however, both islands will need to put the right infrastructure in place to enable a rapid response to an outbreak of any new emergent virus in the future.

## Introduction

Zika virus (ZIKV) is an arbovirus that belongs to the *Flaviviridae* family, genus Flavivirus, and is transmitted through the bite of infected *Aedes aegypti* mosquitoes, via sexual contact (1–3), or from mother to fetus (4). ZIKV infection is often asymptomatic or otherwise presents with mild symptoms such as fever, macopapular rash, conjunctivitis, myalgia, and headache (5). In a small number of cases, ZIKV infection can result in serious complications such as Guillain-Barré syndrome (6–10), maculopathy (11–13), or microcephaly in newborns when the mother is infected with the virus during pregnancy (14–18).

Historically, since its discovery in Uganda in 1947, ZIKV was confined to Africa resulting only in sporadic cases of mild disease. In 2007, however, this pattern changed when the first major outbreak of ZIKV occurred in Yap (Federal States of Micronesia) where ~73% of the population was infected and symptomatic disease developed in ~18% of infected persons (19). Since then, ZIKV spread rapidly across the Pacific Ocean, causing outbreaks in French Polynesia (20), Cook Islands (20), Easter Island (21), New Caledonia (22), until eventually emerging in the Americas (23). Here it was first reported in Brazil in continental South America in May 2015, after which the virus spread to other Latin American countries, such as Colombia (October 2015), Surinam, El Salvador, Mexico, Guatemala, Paraguay, Venezuela (November 2015), Panama, Honduras, French Guiana, Martinique, Puerto Rico (December 2015), Maldives, Guyana, Ecuador, Barbados, Bolivia, Haiti, Saint Martin, Dominican Republic, Nicaragua, Jamaica, Curaçao, Costa Rica (January 2016), Bonaire and Aruba (February 2016) (24, 25). In Brazil alone, it has been estimated that between 440,000 and 1.3 million persons were infected with ZIKV in 2015 (26), and around 2366 cases of ZIKV-associated microcephaly/CNS malformations have been reported (as of February 2017, www.statista.com). Since then, the epidemic continued to spread, and the total number of infected persons and children with congenital ZIKV syndrome still remains to be determined.

Curaçao, a nation of almost 150,000 inhabitants, is known for its circulation of *A. aegypti* transmitted viruses, such as dengue virus (DENV) and chikungunya virus (CHIKV). DENV has been endemic on Curaçao for decades and outbreaks of the virus occur here every few years. The most recent outbreak of dengue occurred in 2014, where Curaçao health authorities had reported 194 suspected and 20 confirmed cases of dengue at the end of August (27). In June of the same year, the first case of CHIKV was also reported, which was the start of a major outbreak on the island that lasted until February 2015. By the end of November 2014, 1,838 suspected and 835 confirmed cases of CHIKV had been reported (28). Dengue is also endemic on Bonaire, a nation with almost 19,000 inhabitants, but not many reports are available.

Due to the high degree of serological cross-reactivity between flaviviruses, confirmation of infection poses a challenge. As IgM is thought to be more specific than IgG, detection of IgM against ZIKV by ELISA represents a possibility; however, cross-reactivity of DENV and ZIKV IgM has been demonstrated (29). This means that confirmative neutralization assays would still be required. As a result, confirmation of flavivirus infections is mostly based on detection of viral RNA in serum by using quantitative real-time PCR (qRT-PCR). However, for several arboviruses such as DENV or ZIKV, the level of viremia present in the blood during the symptomatic phase is often very low, which makes detection problematic. The use of urine as an alternative matrix for detecting ZIKV RNA was investigated by several laboratories and was found to be a good alternative to serum, EDTA-plasma and saliva, due to the higher levels of RNA found, and the longer period of time that urine was found positive after the onset of symptoms (>10–20 days) (30, 31). In contrast, another study demonstrated detection of ZIKV in whole blood for a longer period of time compared to urine and serum (32). Based on these observations, official World Health Organization (WHO) interim recommendations included using either whole blood, serum, or urine for nucleic acid testing (NAT), and serum for IgM detection (33). The routine confirmation of serological results by virus neutralization assays was not recommended as it was considered unfeasible.

To define the scope of the ZIKV outbreak on Curaçao and Bonaire, we determined the number of confirmed ZIKV cases based on qRT-PCR diagnostics, the incidence rates of infection in patients in terms of age and gender, as well as the geospatial distribution of ZIKV cases on Curaçao. In addition, paired urine samples from Curaçao were assessed for ZIKV shedding kinetics, while paired urine and serum samples from Bonaire were compared for sensitivity of ZIKV RNA detection.

## Methods

In the period of 16 December 2015 until 26 April 2017, serum, EDTA-plasma or urine samples were taken at Medical Laboratory Services (MLS) from patients on Curaçao presenting with symptoms resembling ZIKV infection, such as fever, rash, headache or conjunctivitis. Between 16 December 2015 and 15 October 2016, the collected samples were inactivated and stabilized in MagnaPure lysis buffer (Roche Diagnostics, Almere, the Netherlands) and shipped to the diagnostic laboratory of the Erasmus Medical Centre (EMC) in Rotterdam, the Netherlands, where ZIKV RNA was tested by an ISO15189:2012 validated, internally controlled lab-developed real-time semi-quantitative qRT-PCR. In short, total nucleic acids were isolated using an external lysis protocol on the MagNA Pure LC robotics system (Roche Diagnostics) and subsequently tested in two independent qRT-PCRs using TaqMan® 1-Step Fast-Virus Master Mix (Thermo Fisher Scientific, Bleiswijk, the Netherlands) and primers targeting the envelope and the NS2A, in multiplex with an internal control (PDV), in a LC480-II cycler (Roche Life Science) (Table 1). The cut-off was set at <45 *C*_T_ values. Starting from 6 July 2016, only the primer pair targeting the envelope was used in the qRT-PCR for confirmation of ZIKV infection.

**Table 1:** Lab-developed qRT-PCR primers and probe used for diagnostics of ZIKV.

**Name**	**Sequence (5^**′**^-3^**′**^)**	**Conc. (nM)**	**Target**	**PCR product (bp)**	**References**
ZIKV_1086	CCGCTGCCCAACACAAG	600	Envelope	77	(29)
ZIKV_1107	FAM-AGCCTACCTTGACAAGCAGTCAGACACTCAA-BHQ1	100			
ZIKV_1162c	CCACTAACGTTCTTTTGCAGACAT	600			
Zika2_fwd	CTTGGAGTGCTTGTGATT	600	NS2A	187	(34)
Zika2_ probe	FAM-AGAAGAGAATGACCACAAAGATCA-BHQ1	100			
Zika2_rev	CTCCTCCAGTGTTCATTT	600			
PDV fwd	CGGGTGCCTTTTACAAGAAC	600	Heamagglutinine	78	(35)
PDV probe	Cy5-ATGCAAGGGCCAATT-MGB	200			
PDV rev	TTCTTTCCTCAACCTCGTCC	150			

*PDV, phocine distemper virus (internal control); BHQ, black hole quencher*.

As the etiology of the clinical manifestations of patients was still uncertain during the first 2 months of the outbreak (December 2015 and January 2016), serum samples collected from patients were also tested for DENV and CHIKV RNA using FTD Dengue/Chik multiplex (Fast Track Diagnostics, Esch-sur-Alzette, Luxembourg). Between December 2015 and October 2016 paired urine samples (plasma if urine was not available) with a target interval of ~2 weeks were submitted for testing. Starting from February 2016, either urine (matrix of choice) or EDTA-plasma samples (if urine was not available) were collected from patients.

In the period of 17 October 2016 until 26 April 2017, after the implementation of commercial ZIKV diagnostic assays at MLS, all samples of suspected ZIKV patients on Curaçao were collected and tested at MLS. In this period, samples were also collected from ZIKV-suspected patients on Bonaire by Fundashon Mariadal and sent to MLS for testing. In contrast to Curaçao, here it was chosen to collect paired serum and urine samples on the same day, from a large number of patients. The ZIKV diagnostic tests consisted of qRT-PCR and/or IgM/IgG ELISA (Euroimmun, Lübeck, Germany). For qRT-PCR, total nucleic acids were isolated using the MagNA Pure robotics system (Roche Diagnostis) and tested in a qRT-PCR using FTD Zika virus multiplex (Fast Track Diagnostics). Depending on the number of days after the onset of symptoms at which the patient was submitted for testing, the choice was made for either qRT-PCR alone (0–7 days), qRT-PCR and serology (7–14 days), or serology alone (≥14 days). However, as neither an ELISA-positive IgM or IgG result for ZIKV in a DENV-endemic area can be considered reliable due to the cross-reactivity known to exist between DENV and ZIKV antibodies (36, 37), we only considered positive results obtained in the qRT-PCR for the analyses.

In the period of 17 October 2016 up to 8 November 2016, plasma samples were tested, but from 9 November 2016 onwards, serum was chosen over EDTA-plasma due to its superior practicality and durability in the lab, and recommendations made by the World Health Organization (33). Urine was no longer the matrix of choice as serum could be used in both qRT-PCR and ELISA.

Curaçao can be divided into 65 geozones, which consist of one or more neighborhoods. The patients' geozone of residence was used as a proxy for location and plotted on a map of Curaçao using www.mapcustomizer.com. The ZIKV incidence rates were determined for different age categories and the gender of patients.

Information such as presenting symptoms, day of onset, and pregnancy was not properly documented by the general practitioners on either Curaçao or Bonaire, and as a result, this data could not be included in the analyses. Written consent was provided by each individual submitting a urine, serum or plasma sample for testing, and written consent for children under 16 years of age was provided by their parent or guardian. As samples of patients were only collected for diagnostic purposes, no additional ethical clearance was required for this study.

## Statistical Analyses

Paired samples were analyzed using a two-tailed paired *t*-test and *P*-values equal to or less than 0.5 were considered to be statistically significant.

## Results

Between 16 December 2015 and 26 April 2017, 3,833 samples of 2,820 individuals were collected by MLS on Curaçao. Of these, 2,044 samples belonging to 1,685 patients were tested in qRT-PCR, resulting in 815 qRT-PCR positive samples, consisting of 781 positive ZIKV patients (Table 2). Testing of serum samples of ZIKV-suspected patients on Curaçao was first initiated in December 2015, during which two patients tested positive for ZIKV using qRT-PCR. During the first 2 months of the outbreak (December 2015 and January 2016), when serum samples were also tested in DENV and CHIKV qRT-PCRs, four out of 87 ZIKV-suspected disease cases were confirmed as positive for DENV instead (*C*_T_ 26.6, 14.5, 29.5, and 34.4).

**Table 2:** Number of samples collected and tested, and the number of patients tested in qRT-PCR during the ZIKV outbreak on Curaçao and Bonaire.

	**Curaçao**	**Bonaire**
No. of samples collected	3,833	744
No. of patients	2,820	382
No. of samples tested in qRT-PCR	2,044	599
No. of patients tested in qRT-PCR	1,685	358
No. of qRT-PCR positive samples	815	129
No. of qRT-PCR positive patients	781	112
No. of patients submitting paired samples for qRT-PCR	324	–
No. of patients testing qRT-PCR positive for first sample	70	–
No. of patients testing qRT-PCR positive for second sample	32	–
No. of patients submitting paired urine and serum samples	–	262
No. of patients submitting paired urine and serum samples for qRT-PCR	–	183
No. of patients testing qRT-PCR positive for urine only	–	18
No. of patients testing qRT-PCR positive for serum only	–	17
No. of patients testing qRT-PCR positive for both urine and serum	–	13
No. of patients testing qRT-PCR negative for both urine and serum	–	135

**Figure 1:**
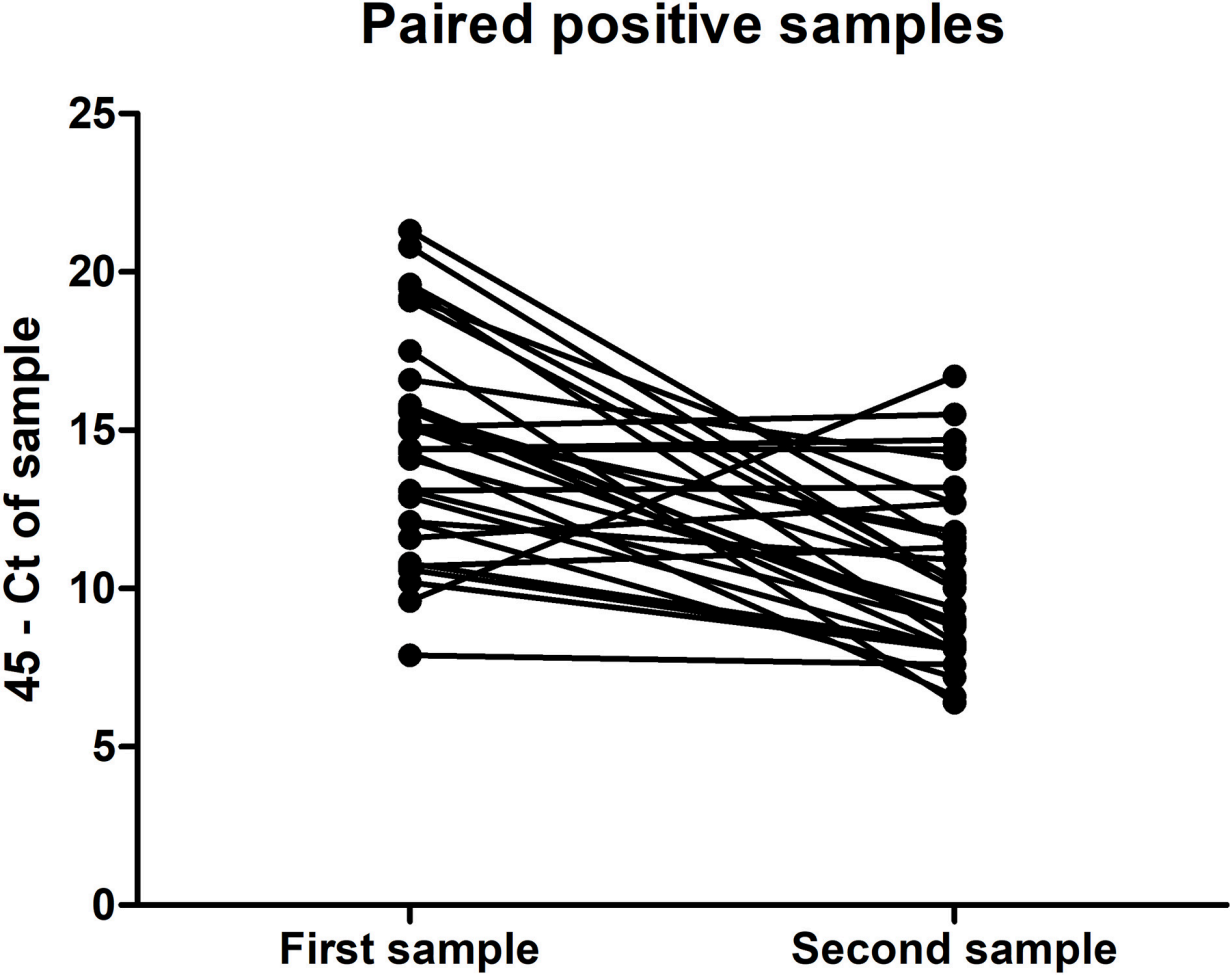
Amount of ZIKV RNA detected in the first and second ZIKV-positive urine samples of the paired samples submitted for testing by 32 individuals, expressed in terms of *C*_T_ threshold 45 minus the *C*_T_ determined for the sample.

Of the 324 patients that submitted paired urine samples between December 2015 and October 2016, 70 patients tested positive for their first sample, while only 32 people still tested qRT-PCR-positive for their second sample (Table 2), taken between 11 and 17 days after the initial sample. This indicates that for some patients in this cohort, ZIKV RNA was still detectable in urine for up to 17 days. Furthermore, there was a significant trend in the decrease in the amount of virus shed in the urine over this time period (*P* < 0.0001, paired *t*-test) (Figure 1). No significant differences were found in the amount of virus shed in the urine between men and women (data not shown).

During the period of 17 October 2016 until 26 April 2017, a total of 744 samples were also collected from 382 individuals on Bonaire and tested at MLS Curaçao. Of these, 599 samples belonging to 358 patients were tested by qRT-PCR. A total of 129 samples consisting of 112 patients tested qRT-PCR positive for ZIKV. Of the 262 patients that had both a serum and urine sample taken on the same day, 183 had both samples concomitantly tested in qRT-PCR. Of these, 13 patients were positive for both serum and urine, while 17 patients tested positive for only serum, and 18 for only urine. One hundred 35 patients tested negative for both (Table 2).

**Figure 2:**
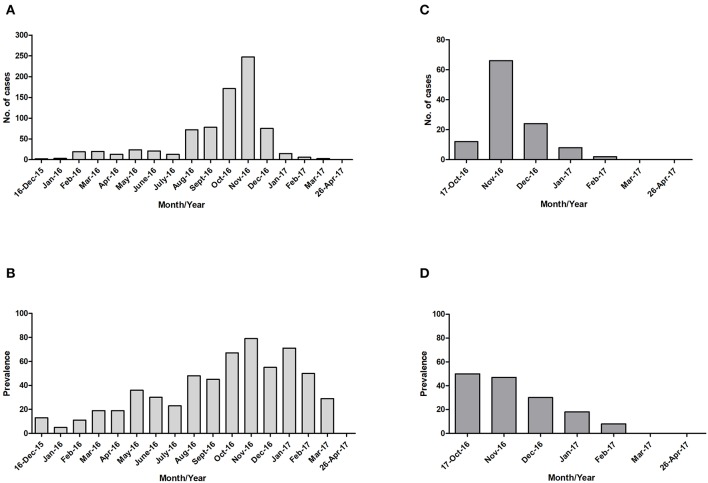
Absolute number of cases and prevalence of ZIKV-positive patients confirmed by qRT-PCR on Curaçao from mid-December 2015 to late April 2017 **(A,B)**, and on Bonaire from mid-October 2016 to late April 2017 **(C,D)**.

For both islands, the peak number of absolute cases occurred in November 2016, with 247 qRT-PCR confirmed cases on Curaçao (Figure 2A) and 66 qRT-PCR-positive cases on Bonaire (Figure 2C; Table 3). In terms of prevalence, for Curaçao, the peak (79%) also occurred in November 2016 (Figure 2B), whereas for Bonaire the peak prevalence (50%) was in October 2016 (Figure 2D; Table 3). Overall, a higher proportion of women than men was diagnosed (~73%) on both Curaçao and Bonaire (Table 4), with incidence rates of 737 and 875 per 100,000, respectively. Furthermore, ZIKV was diagnosed mostly in individuals in the age category of 25–54 years old on both Curaçao (61%; incidence rate of 863 per 100,000) and Bonaire (67%; incidence rate of 815 per 100,000) (Table 4).

**Table 3:** Prevalence per month of qRT-PCR-confirmed ZIKV-positive patients on Curaçao and Bonaire during the outbreak.

**Month**	**No. of patients tested**	**No. of positive patients**	**Prevalence (%)**	**No. of patients tested**	**No. of positive patients**	**Prevalence (%)**
**Curaçao**				**Bonaire**		
16-Dec-15	15	2	13			
Jan-16	66	3	5			
Feb-16	176	19	11			
Mar-16	104	20	19			
Apr-16	68	13	19			
May-16	66	24	36			
June-16	69	21	30			
July-16	56	13	23			
Aug-16	151	72	48			
Sept-16	172	78	45			
Oct-16^*^	254	171	67	24	12	50
Nov-16	311	247	79	141	66	47
Dec-16	136	75	55	79	24	30
Jan-17	21	15	71	45	8	18
Feb-17	12	6	50	26	2	8
Mar-17	7	2	29	31	0	0
26-Apr-17	1	0	0	12	0	0

* *For Bonaire samples were collected starting from 17-Oct-16*.

**Table 4:** Characteristics of the 781 patients confirmed by qRT-PCR for ZIKV infection on Curaçao between 16 December 2015 till 26 April 2017, and of the 112 patients confirmed on Bonaire between 17 October 2016 till 26 April 2017, according to sex and age [with use of population demographics data from July 2017 (www.indexmundi.com)].

**Characteristics**	***N***	**%**	**Population (*N*)**	**Incidence per 100,000 population**
**Curaçao**				
**Total population**	149,648			
**ZIKV positive**	781			
**Sex**				
Female	574	73.5	77,920	737
Male	207	26.5	71,728	289
**Age group**				
0-14	70	9.0	29,935	234
15-24	67	8.6	21,450	312
25-54	476	60.9	55,181	863
55-64	106	13.6	20,482	518
65+	62	7.9	22,600	274
**Bonaire**				
**Total population**	19,179			
**ZIKV positive**	112			
**Sex**				
Female	81	72.3	9261	875
Male	31	27.7	9918	313
**Age group**				
0-14	6	5.4	3,359	179
15-24	15	13.4	2,105	713
25-54	75	67.0	9,198	815
55-64	11	9.8	2,552	431
65+	5	4.5	2,194	228

**Figure 3:**
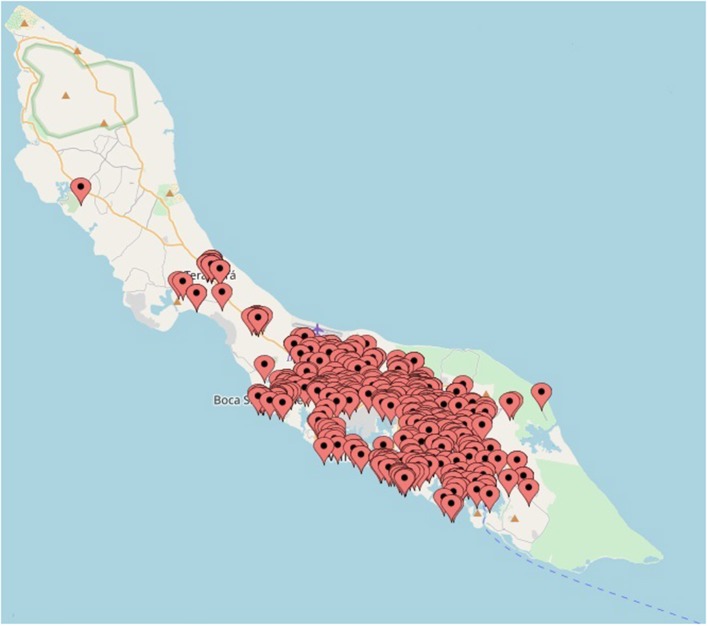
The locations of a selection of the patients on Curaçao that tested positive for ZIKV by qRT-PCR. The map was created by plotting the locations on www.mapcustomizer.com.

To determine the distribution of ZIKV infections on Curaçao, the locations of patients that tested positive for ZIKV by qRT-PCR were plotted on a map of Curaçao. Locations of 197 patients could not be pinpointed on the map. The map shows that the majority of the ZIKV cases were clustered in the eastern part of the island, particularly in Willemstad (Figure 3). Geozones with a notable number of infections included Santa Rosa, Spaanse Water, St. Michiel, Dominguito, Brievengat, Berg Altena, Tera Cora, Stenen Koraal, and Groot Piscadera.

## Discussion

Despite the documented emergence of ZIKV into the Americas in Brazil in May 2015, phylogenetic analyses estimate the introduction of the virus to be earlier, either between August 2013 and July 2014 (38) or between May and December 2013 (39). On Curaçao, according to our analyses, the first cases of ZIKV were diagnosed in December 2015, a month before the first notification to the WHO on 28 January 2016 (24), which indicates that the virus, most likely introduced by travelers, emerged earlier than officially reported. Given the rapid spread of the virus throughout the Americas after its emergence in Brazil, Curaçao, and Bonaire were not prepared for an outbreak of ZIKV, and diagnostic assays had therefore not yet been implemented and validated at MLS. This problem was circumvented by shipping patient samples to the diagnostic laboratory of the EMC in the Netherlands, a WHO Collaborating Centre for arboviruses. Starting from October 2016, MLS Curaçao had implemented the necessary commercial diagnostic qRT-PCR assay and ELISAs in order to continue the diagnosis of ZIKV-suspected patients on Curaçao and start with the diagnostics for Bonaire. Of note, this study was not designed prospectively but performed in reaction to a dynamic outbreak situation.

During this period, a switch was also made from urine to serum for samples collected on Curaçao. Even though a few studies have shown that urine was more sensitive for detection of ZIKV by qRT-PCR compared to serum (30, 31), the data from the paired serum and urine samples from Bonaire suggest that in this cohort, these two matrices were required concomitantly to increase the chance of ZIKV detection. As a result, it is possible that many ZIKV cases on both Curaçao and Bonaire were missed as here, paired urine and serum samples were not consistently collected and/or tested in qRT-PCR. Even though many PCR-negative samples from Curaçao and Bonaire had also been tested in IgM/IgG ELISA, the cross-reactivity known to occur between ZIKV and DENV antibodies makes diagnosis based on serology difficult (36, 37) and could easily lead to false positives. As a result, serology data of samples from patients collected 14 days after onset of symptoms were not included in our analyses, and our results are therefore very likely an underrepresentation of the number of ZIKV cases on both islands. Another factor that may have led to an underrepresentation of the total number of cases is the fact that not all individuals that experienced symptoms went to the general practitioner to get tested. Furthermore, on Curaçao, three laboratories were involved in the diagnostic testing of ZIKV patients, namely MLS, Analytisch Diagnostisch Centrum (ADC) and Laboratorio de Medicos (LabdeMed). If all the data were to be combined, the total number of ZIKV cases would likely be much larger than presented in this article.

The peak of the ZIKV outbreak on Curaçao appeared to occur in November 2016, both in terms of the absolute number of cases and prevalence. For Bonaire, the peak in the absolute number of cases seemed to occur in November 2016 as well, while in terms of prevalence it appeared to occur in October 2016. However, as no ZIKV diagnostics was carried out for Bonaire between mid-December 2015 and mid-October 2016, the data from October is not reliable for comparison with the other months, and it can also not be excluded that a larger number of people on Bonaire may have been infected in one of the months preceding November.

Interestingly, during the reported outbreak of ZIKV on Curaçao and Bonaire, no cases of microcephaly or fatalities due to ZIKV were reported. However, assuming a similar microcephaly risk of 0.02% for pregnant women as calculated for Brazil (40), and a fertility rate of ~2.1 for Curaçao [based on data from 2011 (41)], which is equivalent to ~2,100 live births per year, this would have given 0.42 cases of microcephaly during the outbreak on Curaçao (which lasted approximately a year). It is therefore not surprising that no cases of ZIKV-related microcephaly were observed in a population of only 150,000 and 19,000 people.

During the outbreak of ZIKV on both Curaçao and Bonaire, almost three times more women than men were infected with the virus. Infections occurred mostly in the age category of 25–54 years old for both men and women. This higher proportion of female infections during a ZIKV outbreak was also reported in Surinam (42) and Rio de Janeiro in Brazil (43). This disproportionate infection rate may be explained by the increased testing of pregnant women due to the concerns about microcephaly and other risks for the unborn babies. However, such a trend was also demonstrated in Rio de Janeiro during a DENV outbreak (43), where women were 30% more likely to be diagnosed with DENV than men. One explanation suggested by this study was that women are more conscientious about their health and therefore more likely to visit a general practitioner. Nonetheless, another possibility, as also speculated upon in the Coelho study (43), is that for ZIKV, a higher amount of male-to-female sexual transmissions occur in comparison to female-to-male transmissions. Infection of females by ZIKV via semen has already been demonstrated (1–3), and even though ZIKV has also been detected in the female genital tract and vaginal secretions (44–47), the ability of the virus to productively infect males via vaginal secretions during sexual intercourse has not yet been demonstrated. Furthermore, the influence of female reproductive hormones on ZIKV replication and transmission should also be investigated, as progestins have recently been shown to promote infection of HIV within the female reproductive tract of non-human primates (48).

In order to obtain an impression of the distribution of the number of ZIKV cases on Curaçao, the locations of the patients were plotted on a map. The majority of the cases were located in the east of the island, which may be the result of a reporting bias caused by a higher population density in the east (Willemstad) (41). Nonetheless, for geozones that contained the largest amount of ZIKV cases, no particular trend in terms of population density or average gross monthly income per household was identified (data not shown). It is possible that the geospatial distribution of ZIKV cases is a reflection of the presence of ZIKV-infected mosquitoes; however, as many inhabitants of Curaçao travel to different parts of the island on a daily basis, it is not possible to determine with certainty the location of transmission. Besides mosquito transmission, sexual transmission of ZIKV may also have influenced the geospatial distribution of cases on the island.

## Conclusions

As Curaçao and Bonaire are (potential) hot-spots for emerging and re-emerging arbovirus infections, it is important that the islands are prepared for future outbreaks by implementing the appropriate diagnostic tools in advance. However, in addition to effective diagnostics, it is imperative that the right infrastructure is also put in place to allow communication during an outbreak setting and to facilitate the implementation of risk-reduction activities in order to deal with any infectious disease that may emerge in the future.

## Data Availability Statement

The datasets generated and/or analyzed during the current study are not publicly available due to patient privacy rights but a selection of datasets are available from the corresponding author on reasonable request.

## Ethics Statement

Written consent was obtained from each individual that provided urine, serum or plasma samples. Consent for children under 16 years of age was provided by their parent or guardian. As MLS and the department of Viroscience are mandated to provide laboratory support for outbreak investigations, no additional ethical clearance was sought out.

## Author Contributions

RW, SP, and GB coordinated and supervised the laboratory diagnostics and logistics. SL, SP, and GB were involved with the analyses. SL, SP, MK, and BM wrote the manuscript.

### Conflict of Interest

The authors declare that the research was conducted in the absence of any commercial or financial relationships that could be construed as a potential conflict of interest.

## Abbreviations

ZIKV: Zika virus
DENV: dengue virus
CHIKV: chikungunya virus
IgM: immunoglobulin M
IgG: immunoglobulin G
ELISA: enzyme-linked immunosorbent assay
qRT-PCR: quantitative real-time polymerase chain reaction
RNA: ribonucleic acid
EDTA: ethylenediaminetetraacetic acid
WHO: World Health Organization
NAT: nucleic acid testing
MLS: Medical Laboratory Services
EMC: Erasmus Medical Center
NS2A: non-structural protein 2A
PDV: phocine distemper virus
*C*_T_: cycle threshold
FTD: Fast Track Diagnostics
ADC: Analytisch Diagnostisch Centrum
HIV: human immunodeficiency virus.
